# Supplementary material for: Resolving the full spectrum of human genome variation using Linked-Reads
Source: Genome Res. 2019 Apr;29(4):635–45. doi: 10.1101/gr.234443.118 (PMC6442396; doi:10.1101/gr.234443.118)
Supplement: Supplemental Material [file supp_gr.234443.118_Supplemental_longranger-2.2.zip › longranger-2.2.tar.bz2!/lr2.2-public/lib/stan/cmdstan/stan/doc/api/html/agrad_2fwd_2matrix_2columns__dot__product_8hpp_source.html]

Stan: src/stan/agrad/fwd/matrix/columns\_dot\_product.hpp Source File


|  |  |
| --- | --- |
|  | Stan  2.5.0  probability, sampling & optimization |


- Main Page
- Related Pages
- Namespaces
- Classes
- Files

- File List
- File Members

All Classes Namespaces Files Functions Variables Typedefs Enumerations Enumerator Friends Macros Pages

- src
- stan
- agrad
- fwd
- matrix

columns\_dot\_product.hpp

Go to the documentation of this file.

1 #ifndef STAN\_\_AGRAD\_\_FWD\_\_MATRIX\_\_COLUMNS\_\_DOT\_PRODUCT\_HPP

2 #define STAN\_\_AGRAD\_\_FWD\_\_MATRIX\_\_COLUMNS\_\_DOT\_PRODUCT\_HPP

3

4 #include <vector>

5 #include <stan/math/matrix/Eigen.hpp>

6 #include <stan/math/matrix/typedefs.hpp>

7 #include <stan/math/error\_handling/matrix/check\_matching\_dims.hpp>

8 #include <stan/agrad/fwd/matrix/typedefs.hpp>

9 #include <stan/agrad/fwd/matrix/dot\_product.hpp>

10 #include <stan/agrad/fwd/fvar.hpp>

11

12 namespace stan {

13  namespace agrad {

14

15  template<typename T, int R1,int C1,int R2, int C2>

16  inline

17  Eigen::Matrix<fvar<T>, 1, C1>

18  columns\_dot\_product(const Eigen::Matrix<fvar<T>, R1, C1>& v1,

19  const Eigen::Matrix<fvar<T>, R2, C2>& v2) {

20  stan::math::check\_matching\_dims("columns\_dot\_product(%1%)",v1,"v1",

21  v2,"v2",(double\*)0);

22  Eigen::Matrix<fvar<T>, 1, C1> ret(1,v1.cols());

23  for (size\_type j = 0; j < v1.cols(); ++j) {

24  Eigen::Matrix<fvar<T>,R1,C1> ccol1 = v1.col(j);

25  Eigen::Matrix<fvar<T>,R2,C2> ccol2 = v2.col(j);

26  ret(0,j) = dot\_product(ccol1, ccol2);

27  }

28  return ret;

29  }

30

31  template<typename T, int R1,int C1,int R2, int C2>

32  inline

33  Eigen::Matrix<fvar<T>, 1, C1>

34  columns\_dot\_product(const Eigen::Matrix<fvar<T>, R1, C1>& v1,

35  const Eigen::Matrix<double, R2, C2>& v2) {

36  stan::math::check\_matching\_dims("columns\_dot\_product(%1%)",v1,"v1",

37  v2,"v2",(double\*)0);

38  Eigen::Matrix<fvar<T>, 1, C1> ret(1,v1.cols());

39  for (size\_type j = 0; j < v1.cols(); ++j) {

40  Eigen::Matrix<fvar<T>,R1,C1> ccol1 = v1.col(j);

41  Eigen::Matrix<double,R2,C2> ccol = v2.col(j);

42  ret(0,j) = dot\_product(ccol1, ccol);

43  }

44  return ret;

45  }

46

47  template<typename T, int R1,int C1,int R2, int C2>

48  inline

49  Eigen::Matrix<fvar<T>, 1, C1>

50  columns\_dot\_product(const Eigen::Matrix<double, R1, C1>& v1,

51  const Eigen::Matrix<fvar<T>, R2, C2>& v2) {

52  stan::math::check\_matching\_dims("columns\_dot\_product(%1%)",v1,"v1",

53  v2,"v2",(double\*)0);

54  Eigen::Matrix<fvar<T>, 1, C1> ret(1,v1.cols());

55  for (size\_type j = 0; j < v1.cols(); ++j) {

56  Eigen::Matrix<double,R1,C1> ccol = v1.col(j);

57  Eigen::Matrix<fvar<T>,R2,C2> ccol2 = v2.col(j);

58  ret(0,j) = dot\_product(ccol, ccol2);

59  }

60  return ret;

61  }

62  }

63 }

64 #endif

check\_matching\_dims.hpp

typedefs.hpp

stan::agrad::fvar

**Definition:** fvar.hpp:13

typedefs.hpp

stan::math::check\_matching\_dims

bool check\_matching\_dims(const char \*function, const Eigen::Matrix< T1, R1, C1 > &y1, const char \*name1, const Eigen::Matrix< T2, R2, C2 > &y2, const char \*name2, T\_result \*result)

**Definition:** check\_matching\_dims.hpp:15

dot\_product.hpp

stan::agrad::size\_type

Eigen::Matrix< double, Eigen::Dynamic, Eigen::Dynamic >::Index size\_type

**Definition:** typedefs.hpp:14

stan::agrad::dot\_product

fvar< T > dot\_product(const Eigen::Matrix< fvar< T >, R1, C1 > &v1, const Eigen::Matrix< fvar< T >, R2, C2 > &v2)

**Definition:** dot\_product.hpp:20

fvar.hpp

Eigen.hpp

stan::agrad::columns\_dot\_product

Eigen::Matrix< fvar< T >, 1, C1 > columns\_dot\_product(const Eigen::Matrix< fvar< T >, R1, C1 > &v1, const Eigen::Matrix< fvar< T >, R2, C2 > &v2)

**Definition:** columns\_dot\_product.hpp:18


---

[ Stan Home Page ]

*© 2011–2014,
Stan Development Team.*
